# Mosquito Exposure Risks in Equine Facilities: An Environmental–Managerial Assessment in Western Romania

**DOI:** 10.3390/microorganisms13112637

**Published:** 2025-11-20

**Authors:** Paula Nistor, Livia Stanga, Andreia Chirila, Vlad Iorgoni, Alexandru Gligor, Alexandru Ciresan, Bogdan Florea, Carina Bota, Vlad Cocioba, Ionela Popa, Gabriel Orghici, Ionica Iancu, Cosmin Horatiu Maris, Janos Degi, Viorel Herman

**Affiliations:** 1 Department of Infectious Diseases and Preventive Medicine, Faculty of Veterinary Medicine, University of Life Sciences “King Mihai I” from Timişoara, 300645 Timişoara, Romania; paula.nistor@usvt.ro (P.N.); vlad.iorgoni@usvt.ro (V.I.); alexandru.gligor@usvt.ro (A.G.); carina-maria.bota.fmv@usvt.ro (C.B.); gabriel.orghici@usvt.ro (G.O.); janosdegi@usvt.ro (J.D.); viorel.herman@fmvt.ro (V.H.); 2 Discipline of Microbiology, Faculty of Medicine, “Victor Babes” University of Medicine and Pharmacy, Eftimie Murgu Square 2, 300041 Timişoara, Romania; 3 Department of Surgery, Faculty of Veterinary Medicine, University of Life Sciences “King Mihai I” from Timişoara, 300645 Timişoara, Romania; chirilaandreia@yahoo.com (A.C.); alexandru.ciresan@usvt.ro (A.C.); 4 Department of Internal Medicine, University of Life Sciences “King Mihai I” from Timişoara, 300645 Timişoara, Romania; bogdan-alexandru.florea.fmv@usvt.ro; 5 Department of Animal Husbandry, University of Life Sciences “King Mihai I” from Timişoara, 300645 Timişoara, Romania; vlad-mihai.cocioba.fmv@usvt.ro; 6 Department of Semiology, Faculty of Veterinary Medicine, University of Life Sciences “King Mihai I” from Timişoara, 300645 Timişoara, Romania; ionela.popa@usvt.ro; 7 Department of Forestry, Faculty of Engineering and Applied Technologies, University of Life Sciences “King Mihai I” from Timișoara, 300645 Timișoara, Romania; cosmin.maris@usvt.ro

**Keywords:** West Nile Virus, mosquito exposure, equine facilities, environmental risk factors, zoonotic pathogens, One Health, Romania, risk scoring

## Abstract

West Nile Virus (WNV) is a mosquito-borne zoonosis with recurrent equine and human cases in Romania. Horses, although dead-end hosts, act as sentinels for local viral circulation. Farm-level risk conditions remain under-characterized. This pilot, exploratory cross-sectional study assessed 42 equine facilities in western Romania (2024). A standardized 10-item checklist was applied and a Composite Environmental Risk Score (CERS) (0–10, unweighted) was computed per facility. Spatial analysis in QGIS included distances to nearby water bodies. No serological or entomological data were collected; these are recommended for future validation. Stagnant water occurred at 71.4% (30/42) of facilities, uncovered rain-collecting containers at 64.3% (27/42), and outdoor housing of horses at 81.0% (34/42). Insect screens were present at 21.4% (9/42) and chemical/biological control at 33.3% (14/42). By design, the CERS ranged from 0 to 10; in our sample the observed range was 0–8 because not all assessed risk conditions co-occurred across sites. Overall, 42.9% (18/42) were classified as high risk (≥6). Neurological signs were reported anecdotally by some managers but were not analyzed. Mosquito-favorable conditions are widespread in Romanian equine facilities. CERS shows promise as a low-cost, rapid tool for routine facility-level assessment of environmental conditions favoring mosquito presence and prioritization of preventive actions. Integrating environmental risk scoring with entomological and serological surveillance could strengthen One Health early-warning systems. Such integration would support prevention of WNV and other mosquito-borne zoonotic pathogens in endemic European settings.

## 1. Introduction

West Nile Virus (WNV) is a mosquito-borne zoonotic arbovirus that exemplifies a One Health challenge, as its transmission cycle involves wild birds as reservoirs, mosquitoes as vectors, and incidental mammalian hosts such as humans and horses [1]. It is one of the most widely distributed arboviruses in Europe and remains a recurrent public and veterinary health concern in Romania, where equine and human cases have been reported regularly for over two decades [2,3]. Climatic and environmental changes have facilitated the geographic expansion of WNV, creating multi-sectoral health threats that simultaneously affect human, animal, and ecosystem health [4]. Rising temperatures and altered precipitation patterns create conditions favorable to mosquitoes, accelerating viral amplification and extending the transmission season [5]. Beyond WNV, mosquitoes are also vectors of other arboviruses of veterinary and public health importance in Europe, such as Usutu virus, which underlines the broader relevance of assessing exposure risks at farm level [3]. This exploratory study aims to generate baseline facility-level data to guide future integrated entomological and serological surveillance.

In this ecology, horses play a dual role in WNV epidemiology. Although, like humans, they are dead-end hosts that do not transmit the virus further, WNV infections in equines serve as sentinels signaling local circulation of the pathogen. The appearance of an equine case indicates the presence of infected *Culex* mosquitoes, effectively providing an early warning for potential human exposure [6,7]. Recent studies have highlighted that horses in areas with prior WNV activity show increased seropositivity, supporting their use as indicators of local circulation and the importance of integrating veterinary and public health surveillance within a One Health framework [7,8].

The country harbors a diverse mosquito fauna, with over 50 species documented across seven genera, recently augmented by invasive *Aedes* species such as *Ae. albopictus* and *Ae. japonicus* in urban and peri-urban areas [9,10]. Western Romania, the focus of this study, offers particularly favorable conditions for WNV circulation, including floodplain wetlands, irrigation canals, and pasture water sources that sustain dense vector populations. *Culex pipiens*, the dominant vector, thrives in stagnant water such as ditches, troughs, and poorly drained paddocks, while *Cx*. *modestus* and certain *Aedes* spp. also contribute, particularly in wetland habitats [10,11]. Seasonal WNV activity has been recorded almost every summer since the first major Romanian outbreak in 1996, which caused over 800 confirmed human cases [12]. Although southern and southeastern regions are most affected, western counties such as Timiș, Arad, and Bihor have increasingly reported WNV circulation in both humans and horses [13,14,15].

Extensive entomological and serological surveys have documented WNV exposure in Romanian wildlife, vectors, and domestic animals. Surveillance during the 2000s confirmed that the virus has become endemic in several regions, with recurring detection in wild birds and equines [16]. However, despite this evidence of ongoing circulation, important knowledge gaps remain regarding farm-level risk determinants. Previous seroprevalence studies in horses have provided valuable baseline data and even demonstrated active regional circulation in western Romania and neighboring Bulgaria. However, none assessed how stable conditions, horse management practices, or local ecological factors influence infection risk [14,15]. As a result, it remains unclear why some equine facilities may be more prone to WNV exposure than others. Addressing this gap is crucial for developing targeted interventions. Identifying clusters of equine cases often aligns with environmental “hot spots,” and linking veterinary surveillance with ecological risk indicators could enhance early warning capabilities [7].

To this end, the present study was designed as an exploratory assessment of environmental and management conditions in equine facilities that may create environmental and management conditions that increase mosquito vector density and potential WNV spillover. The Composite Environmental Risk Score (CERS), a standardized 0–10 index, was applied to capture the cumulative presence of mosquito-friendly conditions at each site. To date, no study in Romania has systematically assessed such mosquito-favoring factors at the equine-facility level, and this pilot application of the CERS represents an initial step toward targeted prevention.

In summary, environmental and management mosquito-favoring factors in equine facilities in western Romania were systematically evaluated using a standardized checklist and the CERS, with the aim of identifying practical prevention targets and early-warning, One Health–relevant indicators. By addressing existing knowledge gaps and integrating concepts of vector ecology, One Health surveillance, and on-farm biosecurity, the study offers insights to support more effective strategies for protecting both animal and human health from WNV, with direct relevance to zoonotic risk at the human–animal–environment interface and to One Health early warning.

## 2. Materials and Methods

### 2.1. Study Area and Design

A cross-sectional survey was conducted from May to September 2024 in 42 equine facilities equally distributed across Bihor, Timiș, and Arad counties (14 per county), selected by convenience based on accessibility and willingness to participate. Facilities with fewer than three horses, those under major renovation, or competition premises with restricted access during events were excluded to avoid atypical conditions. Facilities were selected by convenience, based on accessibility, voluntary participation, and fulfillment of inclusion criteria (≥3 horses, permanent activity, absence of major renovation works). Although randomization was not feasible, efforts were made to ensure representation of rural, peri-urban, and wetland-adjacent environments. The threshold of three horses was chosen to ensure epidemiological relevance, since smaller premises may not provide representative management or environmental conditions. The study area encompassed rural, peri-urban, and wetland-adjacent environments with a documented history of WNV activity. Geographic coordinates were recorded with handheld GPS devices and facility locations were mapped and analyzed in QGIS [17,18]. Climatic data from the Romanian National Meteorological Administration indicated mean monthly temperatures of 17–24 °C during the study period, with rainfall sufficient to produce transient standing-water habitats; these temperatures fall within the range known to enhance *Culex* mosquito vectorial capacity [18]. Distances from each facility to the nearest rivers or lakes were calculated in QGIS to account for potential vector habitats. Land cover metrics were not assessed [19].

### 2.2. Environmental Assessment Protocol

Structured field visits were conducted at each facility using a standardized 10-item checklist adapted from validated protocols for equine mosquito risk assessments. The checklist underwent expert review by two veterinary epidemiologists and one medical entomologist and was pilot-tested in two facilities (excluded from analysis) to ensure clarity and inter-observer consistency. Observers assessed multiple factors including (1) presence of stagnant water near stables, (2) open containers collecting rainwater, (3) vegetation density, (4) use of insect screens, (5) mosquito control practices, (6) overnight housing of horses, (7) stable hygiene, (8) waste management, and (9) staff awareness of WNV transmission [20,21]. Stable hygiene was rated by manure removal frequency, ventilation, and flooring maintenance. Waste management referred to manure disposal protocols, collection frequency, and slurry prevention. Facility managers were also asked to report any neurological cases in horses observed over the past two years (item 10). These data were collected only as supplementary contextual information and not as primary study outcomes. All facility owners/managers provided verbal consent prior to each visit [20]. Photographic documentation focused on environmental features that could support mosquito development (e.g., water collections, dense foliage) and served as supplementary records for validation [20]. No active mosquito trapping or larval sampling was performed during the visits; instead, the presence of potential mosquito breeding sites (such as puddles, water troughs, or manure slurry pits) was documented observationally. All field observations represent indirect or proxy indicators of mosquito-favorable conditions. No entomological sampling was performed, thus, the observed features (e.g., puddles, containers, vegetation) should be interpreted as potential, not confirmed, breeding habitats.

### 2.3. Risk Factor Criteria

The ten environmental and management criteria used for facility assessment are summarized in Table 1. Each checklist item corresponded to a yes/no or categorical evaluation of a potential WNV risk factor (e.g., presence of stagnant water, quality of stable hygiene, occurrence of equine neurological cases, etc.), as detailed in Table 1.

### 2.4. Composite Environmental Risk Score (CERS)

For each facility, criteria were scored as 1 (present) or 0 (absent), and all were assumed to contribute equally to the Composite Environmental Risk Score (CERS). All ten criteria were applied consistently across all facilities. Scores were summed to yield a total ranging from 0 to 10, with facilities classified as Low risk (0–2), Moderate risk (3–5), or High risk (≥6). The threshold for ‘High risk’ (CERS ≥ 6) was established empirically, corresponding to the presence of at least 60% of unfavorable conditions, and ensuring consistency with previously published equine mosquito risk assessment protocols. An unweighted composite scoring system was applied, consistent with previous equine mosquito risk assessment protocols [20,22], in order to ensure simplicity, transparency, and comparability across facilities. It is acknowledged that this approach may underweight certain high-impact factors.

### 2.5. Statistical Analysis

Descriptive statistics were computed for all variables. Proportions for each risk factor and for CERS categories were estimated with Wilson 95% confidence intervals (CI) [23,24]. Geographic patterns were explored by comparing the distribution of CERS categories among the three counties using χ^2^ tests for trend. Exploratory associations were assessed using odds ratios (ORs) and logistic regression, with outcome High CERS (≥6) versus <6 and predictors such as stagnant water, lack of insect screens, and outdoor housing. ORs with 95% CIs were reported; α = 0.05, two-tailed. All analyses were performed in IBM SPSS Statistics v29 (IBM Corp., Armonk, NY, USA) [25]. No clinical or serological data were analyzed. Given the pilot nature and small sample size, only descriptive and exploratory statistical tests were applied. Future expanded analyses will incorporate spatial statistics (e.g., Moran’s I for spatial autocorrelation) and multivariable models treating proximity to water bodies as a continuous predictor.

## 3. Results

### 3.1. Environmental and Managerial Mosquito-Favoring Factors

**Figure 1:**
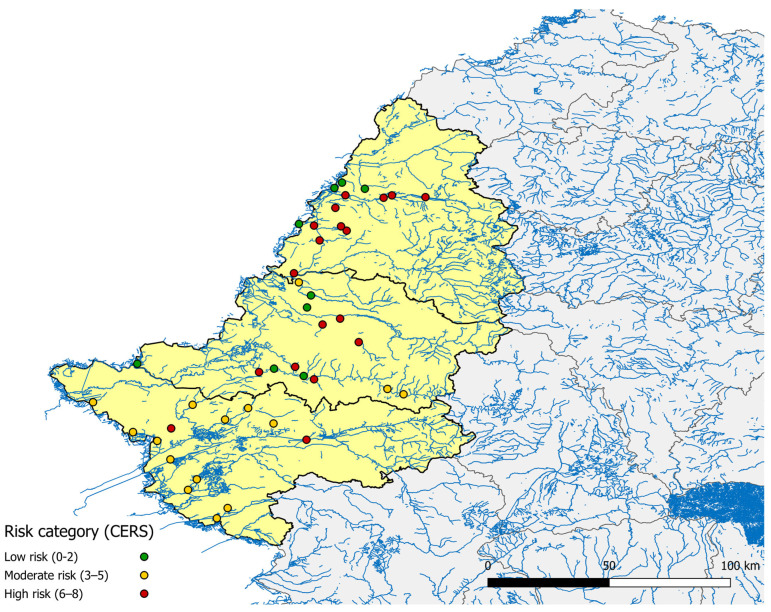
Geographical distribution of the 42 surveyed equine facilities across Bihor, Arad, and Timiș counties in western Romania, categorized by Composite Environmental Risk Score (CERS): Low (0–2), Moderate (3–5), High (≥6).

A total of 42 equine facilities were surveyed across the three counties (Bihor, Arad, and Timiș) in western Romania, with their locations and corresponding CERS mapped in Figure 1.

**Figure 2:**
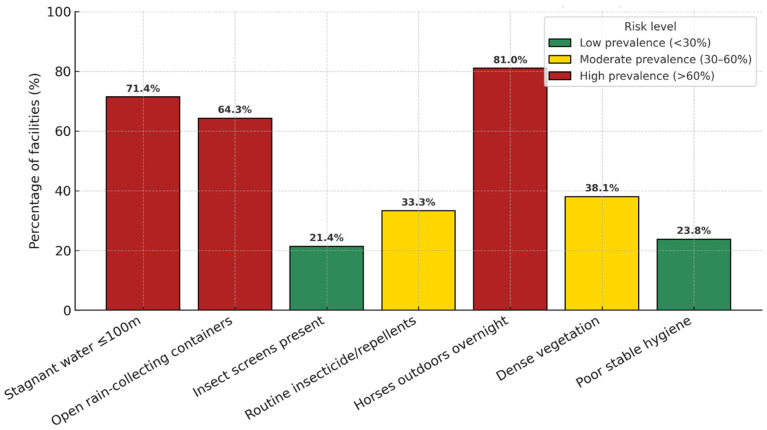
Prevalence of environmental and managerial mosquito-favoring factors favoring mosquito exposure in equine facilities from western Romania (n = 42).

Key environmental and managerial indicators are summarized in Table 2. The distribution of these mosquito-favoring factors is further illustrated in Figure 2, which shows their relative prevalence across facilities.

The bar chart shows the proportion of facilities where each factor was observed. Risk levels are color-coded as follows: green (<30%, low prevalence), yellow (30–60%, moderate prevalence), and red (>60%, high prevalence). The most frequent factors were horses kept outdoors overnight (81.0%), stagnant water within 100 m of stables (71.4%), and uncovered rain-collecting containers (64.3%), while protective measures such as insect screens (21.4%) and routine insecticide use (33.3%) were less common.

By design, the Composite Environmental Risk Score (CERS) spans 0–10. In this dataset, observed values ranged from 0 to 8 because not all assessed risk conditions co-occurred at any single facility. Overall, 21.4% of facilities were classified as low risk (0–2), 35.7% as moderate (3–5), and 42.9% as high risk (≥6). In exploratory logistic models, the absence of insect screens and the presence of stagnant water showed positive but imprecise associations with high CERS (≥6); confidence intervals were wide and not statistically significant.

**Figure 3:**
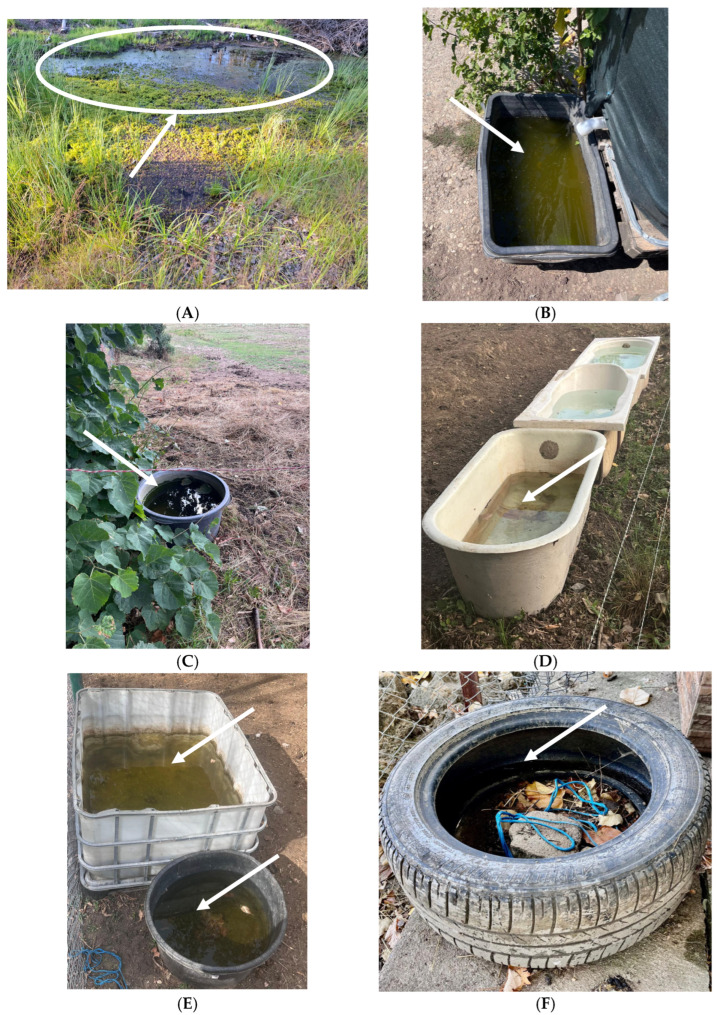
Examples of environmental conditions observed in equine facilities: (**A**) temporary puddles; (**B**) stagnant water near stables; (**C**) open containers; (**D**) water troughs; (**E**) deficient drainage; (**F**) outdoor housing. Arrows indicate the key features highlighted.

Natural water bodies within close proximity were identified in 71.4% of facilities, and artificial containers or structures holding water (e.g., troughs, buckets, disused tires) were recorded in 64.3%. Figure 3 provides photographic examples of potential mosquito breeding habitats documented during field visits.

### 3.2. Seroprevalence and Spatial Correlation

No serological testing was performed in the present study. Distances from equine facilities to the nearest rivers or lakes were measured in QGIS. Several facilities in Bihor and Arad counties with high CERS values were located within 1.5 km of major water bodies, suggesting that proximity to natural aquatic habitats may contribute to local mosquito breeding potential.

## 4. Discussion

The present study assessed environmental and managerial conditions that may favor mosquito exposure in equine facilities from western Romania. Several key findings emerged. A high proportion of equine facilities presented mosquito-favorable conditions, such as stagnant water near stables (71.4%), uncovered rain-collecting containers (64.3%), and horses kept outdoors overnight (81.0%). In contrast, mosquito-control measures such as insect screens (21.4%) or regular insecticide application (33.3%) were infrequent. Consequently, nearly half of the facilities (42.9%) were classified as high-risk according to the CERS. Although some facilities anecdotally reported neurological signs, these unverified reports were not analyzed and cannot support any association with CERS.

### 4.1. Interpretation of Findings

The persistence of stagnant water and open containers indicates structural gaps in environmental management at equine facilities. Combined with the widespread practice of keeping horses outdoors overnight, these conditions amplify potential exposure to mosquitoes during peak vector activity. The findings suggest that prevention must shift from reactive measures to proactive habitat management. [26,27]. The low implementation of insect control, either structural (screens) or chemical, reflects both limited awareness among farm managers and the absence of systematic prevention strategies at the farm level [20,28].

The lack of vaccination across all surveyed horses represents an additional vulnerability. Although vaccination does not prevent viral circulation, it reduces the risk of severe disease and could mitigate clinical and economic impacts in equine populations [29,30]. The anecdotal mention of neurological signs underscores the need for future clinical and serological follow-up, which was outside this study’s scope. Previous equine serosurveys in western Romania reported WNV seroprevalence of 7.3–8.9% [14], supporting endemic circulation in the study area. The near-ubiquity of core risks likely attenuated detectable between-facility contrasts, explaining the lack of significant associations.

### 4.2. Practical Implications

From a veterinary and public health perspective, the findings emphasize that relatively simple measures, such as eliminating standing water, improving waste and vegetation management, and using physical barriers or repellents, represent practical preventive strategies that can be prioritized in equine facilities [31]. Horses remain valuable sentinels for early detection of WNV activity, and enhancing farm-level biosecurity not only protects animal health but also provides indirect protection for human communities in endemic areas [8,32,33].

Integrating equine surveillance data with environmental risk indicators, such as those captured by the CERS, can strengthen One Health early warning systems [34,35]. In practice, veterinarians should encourage owners to adopt practical mitigation strategies and report neurological cases promptly. Public health authorities could then use such risk maps to prioritize interventions in high-risk zones. These results could inform national guidelines for equine-facility biosecurity in Romania and neighboring WNV-endemic countries.

### 4.3. Comparison with Other Studies

The findings of this study, which documented widespread mosquito-favorable conditions in equine facilities but no statistically significant association between CERS and neurological cases, can be positioned within a broader international context. In Florida, a case–control study demonstrated the strong protective role of WNV vaccination, while management factors such as stable fans, solid housing structures, and the presence of dead birds were identified as risk indicators [36]. In Andalusia, Spain, horse movements and mosquito presence on farms were linked to seropositivity, whereas in Camargue, France, landscape-level habitats such as rice fields and wetlands were associated with case clusters [37,38].

In Germany, serological surveys identified pony breeds, permanent outdoor housing, and residence in previously affected regions as mosquito-favoring factors [39]. In Egypt, intrinsic host factors such as age and sex were the main predictors of exposure [40]. Complementary entomological surveys in Belgium confirmed that equestrian farms provide highly suitable habitats for WNV vectors, with *Culex pipiens* and *Culex torrentium* dominating larval sites [20].

Recent research from 2024 highlights the ongoing challenges and strategies for controlling mosquito exposure and WNV risk in equine and livestock environments, with a focus on lessons from the USA. While no 2024–2025 studies from Argentina were found, several US-based papers provide relevant context for environmental and managerial risk factors, control measures, and the importance of vaccination and integrated surveillance [41,42,43,44,45,46,47].

Studies confirm that stagnant water, uncovered containers, and outdoor housing of animals are common risk factors for mosquito proliferation in equine and livestock settings, echoing findings from Romania. These environmental gaps, if unaddressed, amplify the risk of WNV and other mosquito-borne diseases. Livestock, including horses, can serve as sentinel species for arbovirus surveillance, and their management directly influences mosquito density and disease risk [42,43,48,49,50,51,52].

Integrated vector management, combining proactive habitat management, physical barriers, and chemical controls, remains the most effective approach. In the USA, equine vaccination has dramatically reduced WNV cases in horses, with outbreaks now mostly limited to unvaccinated populations. However, human vaccination is still unavailable, and public education, surveillance, and novel vector control (e.g., genetically modified mosquitoes) are under active development [41,43,44,53,54,55,56]. Organophosphate insecticides, when used for mosquito control, have not shown significant adverse health effects in recent US studies, supporting their continued use with appropriate safety measures [45,57].

Environmental and managerial risk factors are highly prevalent across regions, but their impact varies with local ecological and management contexts. The One Health approach, integrating animal, human, and environmental health, remains essential for effective surveillance and outbreak prevention [42,43,58,59].

Overall, these comparisons highlight that while environmental and managerial risks were highly prevalent in Romanian facilities, their near ubiquity may explain the absence of significant associations with clinical outcomes. International evidence suggests that the relative importance of vaccination, animal movement, ecological habitats, housing management, and host demographics varies across geographic contexts. This supports the view that WNV prevention strategies must be tailored locally, integrating both farm-level biosecurity and regional ecological characteristics.

### 4.4. Study Limitations

Several limitations should be considered when interpreting these results. The sample size was modest (42 facilities) and based on convenience selection, which may limit the representativeness of findings across the wider horse population of western Romania. Entomological sampling was not performed, so the presence of mosquito larvae or adult vectors was inferred from environmental indicators rather than directly measured. Neurological case history was reported anecdotally by facility managers. When feasible, these reports were cross-checked with attending veterinarians or available records; however, data were incomplete and inconsistent across sites. Consequently, clinical information was treated as supplementary context only and was not included in statistical analyses.

The absence of active entomological data collection (e.g., mosquito trapping, larval sampling, species identification) constitutes a key limitation. The inference of vector presence based solely on environmental indicators may underestimate or overestimate the true exposure risk. Therefore, the term “potential risk” is used throughout the manuscript to emphasize the proxy nature of the observations. Future studies should integrate active entomological and serological surveillance in parallel with facility assessments to validate the CERS and establish direct correlations between mosquito abundance, species composition, and facility-level risk scores.

Another methodological limitation concerns the construction of the CERS. All ten criteria were assigned equal weight, an approach that favors simplicity and transparency but implicitly assumes that each factor contributes equally to potential mosquito exposure. This unweighted design may underrepresent the influence of high-impact factors such as stagnant water or lack of mosquito control. As the pilot study did not include entomological or serological data, the predictive validity of the CERS remains untested. Future work will assess construct validity through correlations between individual CERS components and proxy indicators such as vector density, distance to water bodies, and climatic variables. Multivariate approaches (e.g., logistic regression, principal component analysis) will be applied to determine relative factor weights and develop a weighted CERS model with improved predictive performance.

### 4.5. Future Directions

Future research should incorporate longitudinal monitoring of equine facilities, combined with entomological surveillance, to validate and refine the CERS. Intervention trials (e.g., targeted water management, screens, vaccination) should test CERS responsiveness and outcome impact. Expanding the geographic scope and integrating equine data into national WNV surveillance could enhance the capacity to predict and respond to outbreaks in a One Health framework.

## 5. Conclusions

This pilot study documents widespread environmental and managerial risk conditions favoring mosquito exposure in equine facilities in western Romania. Nearly half of facilities met High CERS (≥6) criteria. CERS can be used as a low-cost, rapid tool for routine risk assessment and prioritization of preventive actions at farm level. Integrating facility-level risk scoring with entomological and serological surveillance can strengthen One Health early-warning systems and support prevention of WNV and other mosquito-borne zoonotic pathogens. However, the CERS tool requires validation with entomological and serological data before large-scale application. Future large-scale surveys encompassing all Romanian regions are currently being planned to validate the CERS model across diverse climatic and management contexts and to ensure broader national representativeness.

## Figures and Tables

**Table 1:** Environmental and management risk factor criteria.

No.	Assessment Criterion	Response Options
1	Presence of stagnant water within 100 m of the stable	Yes/No
2	Presence of open containers collecting rainwater	Yes/No
3	Vegetation density and maintenance status	Dense/Moderate/Well-maintained
4	Use of insect screens in stable buildings	Yes/No
5	Routine use of insect repellents or insecticides	Yes/No (specify type)
6	Horses kept outdoors overnight	Yes/No (Open pasture/Sheltered pen)
7	Stable hygiene and structural maintenance	Good/Moderate/Poor
8	Presence of manure waste management protocols	Yes/No
9	Personnel knowledge about mosquito-borne WNV transmission	Yes/No
10	Neurological clinical cases in horses in past 2 years	None/Suspected/Confirmed

**Table 2:** Key facility-level risk indicators (n = 42), with prevalence and Wilson 95% confidence intervals.

Indicator	n/N	%	95% CI
Stagnant water ≤ 100 m	30/42	71.4	56.4–82.8
Open rain-collecting containers	27/42	64.3	49.2–77.0
Insect screens present	9/42	21.4	11.7–35.9
Routine repellents/insecticides	14/42	33.3	21.0–48.4
Horses outdoors overnight	34/42	81.0	66.7–90.0
Dense/overgrown vegetation	16/42	38.1	25.0–53.2
Poor stable hygiene	10/42	23.8	13.5–38.5

Additional factors documented but not shown in the table include presence of formal waste management protocols in 66.7% of facilities and low awareness of WNV among staff in 38.1%. All surveyed horses were unvaccinated for WNV.

## Data Availability

The original contributions presented in this study are included in the article. Further inquiries can be directed to the corresponding authors.

## Abbreviations

The following abbreviations are used in this manuscript:

WNV	West Nile Virus
IgM	Immunoglobulin M
CERS	Composite Environmental Risk Score

## References

[1] Vargas Campos C.A., García-Pérez S., Figuerola J., la Puente J.M.-D., Polo I., Rodríguez-De-Fonseca B., Fernández-Álvarez S., Fraile V.G., Martín-Rey M., Lacasaña M. (2025). Comprehensive analysis of West Nile Virus transmission: Environmental, ecological, and individual factors. An umbrella review. One Health.

[2] Savuła G., Luanda O., Aniłă A., Aniłă D. (2008). West Nile Virus infections in Romania—Past, present and perspective. Lucr. Științifice Med. Vet. Timișoara.

[3] Simonin Y. (2024). Circulation of West Nile Virus and Usutu Virus in Europe: Overview and Challenges. Viruses.

[4] Wang H.R., Liu T., Gao X., Wang H.B., Xiao J.H. (2024). Impact of climate change on the global circulation of West Nile virus and adaptation responses: A scoping review. Infect. Dis. Poverty.

[5] Gizaw Z., Salubi E., Pietroniro A., Schuster-Wallace C.J. (2024). Impacts of climate change on water-related mosquito-borne diseases in temperate regions: A systematic review of literature and meta-analysis. Acta Trop..

[6] Blitvich B.J., Fernandez-Salas I., Contreras-Cordero J.F., Marlenee N.L., Gonzalez-Rojas J.I., Komar N., Gubler D.J., Calisher C.H., Beaty B.J. (2003). Serologic evidence of West Nile virus infection in horses, Coahuila State, Mexico. Emerg. Infect. Dis..

[7] García-Carrasco J.M., Muñoz A.R., Olivero J., Segura M., García-Bocanegra I., Real R. (2023). West Nile virus in the Iberian Peninsula: Using equine cases to identify high-risk areas for humans. Euro. Surveill..

[8] Gothe L.M.R., Ganzenberg S., Ziegler U., Obiegala A., Lohmann K.L., Sieg M., Vahlenkamp T.W., Groschup M.H., Hörügel U., Pfeffer M. (2023). Horses as sentinels for the circulation of flaviviruses in Eastern-Central Germany. Viruses.

[9] Dinu S., Stancu I.G., Cotar A.I., Ceianu C.S., Pintilie G.V., Karpathakis I., Fălcuță E., Csutak O., Prioteasa F.L. (2024). Continuous and dynamic circulation of West Nile Virus in mosquito populations in Bucharest area, Romania, 2017–2023. Microorganisms.

[10] Horváth C., Cazan C.D., Mihalca A.D. (2021). Emergence of the invasive Asian bush mosquito, Aedes (Finlaya) japonicus japonicus, in an urban area, Romania. Parasites Vectors.

[11] Crivei L.A., Moutailler S., Gonzalez G., Lowenski S., Crivei I.C., Porea D., Anita D.C., Ratoi I.A., Zientara S., Oslobanu L.E. (2023). Detection of West Nile Virus Lineage 2 in Eastern Romania and first identification of Sindbis Virus RNA in mosquitoes analyzed using high-throughput microfluidic real-time PCR. Viruses.

[12] European Centre for Disease Prevention and Control (2011). West Nile Virus Outbreak in Romania, July to October 1996.

[13] Oslobanu L.E., Pâslaru A., Savuța G. (2015). West Nile Virus seroprevalence in horses from Romania: First step in the infection risk assessment. Bull. Univ. Agric. Sci. Vet. Med. Cluj-Napoca Vet. Med..

[14] Nistor P., Stanga L., Chirila A., Iorgoni V., Gligor A., Ciresan A., Popa I., Florea B., Imre M., Cocioba V. (2025). Seroprevalence and passive clinical surveillance of West Nile Virus in horses from ecological high-risk areas in Western Romania: Exploratory findings from a cross-sectional study. Microorganisms.

[15] Kirov P., Iancu I., Panayotova E., Petrov R., Imre M., Herman V., Hristov H., Abudalleh A., Alexandrova R., Gligor A. (2025). First trans-border serological evidence of West Nile Virus infection in horses in Romania and Bulgaria. Int. J. Vet. Sci..

[16] Oslobanu L.E., Mihu-Pintilie A., Aniță D., Aniță A., Lecollinet S., Savuța G. (2014). West Nile virus reemergence in Romania: A serologic survey in host species. Vector Borne Zoonotic Dis..

[17] García-Bocanegra I., Franco J., León C., Barbero-Moyano J., García-Miña M., Fernández-Molera V., Gómez M., Cano-Terriza D., Gonzálvez M. (2022). High exposure of West Nile Virus in equid and wild bird populations in Spain following the epidemic outbreak in 2020. Transbound. Emerg. Dis..

[18] De Heus P., Kolodziejek J., Hubálek Z., Dimmel K., Racher V., Nowotny N., Cavalleri J. (2021). West Nile Virus and Tick-Borne Encephalitis Virus are endemic in equids in Eastern Austria. Viruses.

[19] Calzolari M., Angelini P., Bolzoni L., Bonilauri P., Cagarelli R., Canziani S., Cereda D., Cerioli M.P., Chiari M., Galletti G. (2020). Enhanced West Nile Virus circulation in the Emilia-Romagna and Lombardy Regions (Northern Italy) in 2018 detected by entomological surveillance. Front. Vet. Sci..

[20] Boukraa S., De La Grandière M., Bawin T., Raharimalala F., Zimmer J., Haubruge E., Thiry E., Francis F. (2016). Diversity and ecology survey of mosquitoes potential vectors in Belgian equestrian farms: A threat prevention of mosquito-borne equine arboviruses. Prev. Vet. Med..

[21] Chapman G., Archer D., Torr S., Solomon T., Baylis M. (2016). Potential vectors of equine arboviruses in the UK. Vet. Rec..

[22] Zannoli S., Sambri V. (2019). West Nile Virus and Usutu Virus co-circulation in Europe: Epidemiology and implications. Microorganisms.

[23] National Institute of Standards and Technology (NIST) (2025). Wilson method for calculating confidence intervals for proportions. Engineering Statistics Handbook.

[24] Orawo L.A. (2021). Confidence intervals for the binomial proportion: A comparison of four methods. Open J. Stat..

[25] IBM Corp (2025). IBM SPSS Statistics.

[26] Söderroos D., Ignell R., Andersen H., Bergvall K., Riihimäki M. (2023). The effect of insect bite hypersensitivity on movement activity and behaviour of the horse. Animals.

[27] Honnen A., Kypke J., Hölker F., Monaghan M. (2019). Artificial light at night influences clock-gene expression, activity, and fecundity in the mosquito Culex pipiens f. molestus. Sustainability.

[28] Bergmann F., Trachsel D.S., Stoeckle S.D., Sierra J.B., Lübke S., Groschup M.H., Gehlen H., Ziegler U. (2022). Seroepidemiological survey of West Nile Virus infections in horses from Berlin/Brandenburg and North Rhine-Westphalia, Germany. Viruses.

[29] Long M.T., Gibbs E.P., Mellencamp M.W., Bowen R.A., Seino K.K., Zhang S., Beachboard S.E., Humphrey P.P. (2007). Efficacy, duration, and onset of immunogenicity of a West Nile Virus vaccine, live flavivirus chimera, in horses with a clinical disease challenge model. Equine Vet. J..

[30] Saiz J.C. (2020). Animal and human vaccines against West Nile Virus. Pathogens.

[31] Riccardo F., Bolici F., Fafangel M., Jovanovic V., Socan M., Klepac P., Plavsa D., Vasic M., Bella A., Diana G. (2020). West Nile Virus in Europe: After action reviews of preparedness and response to the 2018 transmission season in Italy, Slovenia, Serbia and Greece. Glob. Health.

[32] Magallanes S., Llorente F., Ruiz-López M.J., la Puente J.M.-D., Soriguer R., Calderon J., Jímenez-Clavero M.Á., Aguilera-Sepúlveda P., Figuerola J. (2023). Long-term serological surveillance for West Nile and Usutu Virus in horses in South-West Spain. One Health.

[33] Leblond A., Lecollinet S. (2017). Clinical screening of horses and early warning for West Nile Virus. Equine Vet. Educ..

[34] Kolodziejek J., Jungbauer C., Aberle S.W., Allerberger F., Bagó Z., Camp J.V., Dimmel K., de Heus P., Kolodziejek M., Schiefer P. (2018). Integrated analysis of human-animal-vector surveillance: West Nile Virus infections in Austria, 2015–2016. Emerg. Microbes Infect..

[35] Petrović T., Šekler M., Petrić D., Lazić S., Debeljak Z., Vidanović D., Ćupina A.I., Lazić G., Lupulović D., Kolarević M. (2018). Methodology and results of integrated WNV surveillance programmes in Serbia. PLoS ONE.

[36] Rios L.M., Sheu J.J., Day J.F., Maruniak J.E., Seino K., Zaretsky H., Long M.T. (2009). Environmental risk factors associated with West Nile Virus clinical disease in Florida horses. Med. Vet. Entomol..

[37] García-Bocanegra I., Arenas-Montes A., Napp S., Jaén-Téllez J.A., Fernández-Morente M., Fernández-Molera V., Arenas A. (2012). Seroprevalence and risk factors associated with West Nile Virus in horses from Andalusia, Southern Spain. Vet. Microbiol..

[38] Leblond A., Sandoz A., Lefebvre G., Zeller H., Bicout D.J. (2007). Remote sensing-based identification of environmental risk factors associated with West Nile disease in horses in Camargue, France. Prev. Vet. Med..

[39] Ganzenberg S., Sieg M., Ziegler U., Pfeffer M., Vahlenkamp T.W., Hörügel U., Groschup M.H., Lohmann K.L. (2022). Seroprevalence and risk factors for equine West Nile Virus infections in Eastern Germany, 2020. Viruses.

[40] Selim A., Megahed A., Kandeel S., Alouffi A., Alshazi M. (2021). West Nile Virus seroprevalence and associated risk factors among horses in Egypt. Sci. Rep..

[41] Cendejas P., Goodman A. (2024). Vaccination and Control Methods of West Nile Virus Infection in Equids and Humans. Vaccines.

[42] Olagunju E., Ayewumi I., Adeleye B. (2024). Effects of Livestock-Keeping on the Transmission of Mosquito-Borne Diseases. Zoonoses.

[43] Naveed A., Eertink L., Wang D., Li F. (2024). Lessons Learned from West Nile Virus Infection: Vaccinations in Equines and Their Implications for One Health Approaches. Viruses.

[44] Dapari R., Fadzil M., Hanzir M., Jais J., Safarudin N., Albar A. (2024). Factors Associated with Mosquito Control among Construction Workers: A Systematic Review. PLoS ONE.

[45] Tai Z., Connelly C., Lange S., Foley N., De J., Rivera L., Lozano S., Nett R. (2024). A Scoping Review to Determine if Adverse Human Health Effects Are Associated with Use of Organophosphates for Mosquito Control. J. Med. Entomol..

[46] Miranda L., Rudd S., Mena O., Hudspeth P., Barboza-Corona J., Park H., Bideshi D. (2024). The Perpetual Vector Mosquito Threat and Its Eco-Friendly Nemeses. Biology.

[47] Campos A., Franco A., Godinho F., Huff R., Candido D., Da Cruz Cardoso J., Hua X., Claro I., Morais P., Franceschina C. (2024). Molecular Epidemiology of Western Equine Encephalitis Virus, South America, 2023–2024. Emerg. Infect. Dis..

[48] García-Suárez O., Tolsá-García M., Arana-Guardía R., Rodríguez-Valencia V., Talaga S., Pontifes P., Machain-Williams C., Suzán G., Roiz D. (2024). Seasonal Mosquito (Diptera: Culicidae) Dynamics and the Influence of Environmental Variables in a Land Use Gradient from Yucatán, Mexico. Acta Trop..

[49] Velde F., Overgaard H., Bastien S. (2024). An Integrated Human Behavioral Model for Mosquito-Borne Disease Control: A Scoping Review of Behavior Change Theories Used to Identify Key Behavioral Determinants. Heliyon.

[50] Mader E., Clements N., Lehane Á., Gangloff-Kaufmann J., Crans S., Horton C., Safi A. (2024). A Qualitative Analysis of Perceived Risks and Benefits of Mosquito Abatement and Bite Prevention Strategies in Northeastern U.S. Communities. J. Med. Entomol..

[51] Al-Eitan L., Alnimri M., Ali H., Alkhawaldeh M., Mihyar A. (2024). Mosquito-Borne Diseases: Assessing Risk and Strategies to Control Their Spread in the Middle East. J. Biosaf. Biosecurity.

[52] Vissani M., Alamos F., Tordoya M., Minatel L., Schammas J., Santos M., Trono K., Barrandeguy M., Balasuriya U., Carossino M. (2024). Outbreak of Western Equine Encephalitis Virus Infection Associated with Neurological Disease in Horses Following a Nearly 40-Year Intermission Period in Argentina. Viruses.

[53] Nejati J., Azari-Hamidian S., Oshaghi M., Vatandoost H., White V., Moosa-Kazemi S., Bueno-Marí R., Hanafi-Bojd A., Endersby-Harshman N., Axford J. (2024). The Monsoon-Associated Equine South African Pointy Mosquito Aedes caballus: The First Comprehensive Record from Southeastern Iran with a Description of Ecological, Morphological, and Molecular Aspects. PLoS ONE.

[54] Wouters R., Beukema W., Schrama M., Biesmeijer K., Braks M., Helleman P., Schaffner F., Van Slobbe J., Stroo A., Van Der Beek J. (2024). Local Environmental Factors Drive Distributions of Ecologically-Contrasting Mosquito Species (Diptera: Culicidae). Sci. Rep..

[55] Ripoll L., Iserte J., Cerrudo C., Presti D., Serrat J., Poma R., Mangione F., Micheloud G., Gioria V., Berrón C. (2025). Insect-Specific RNA Viruses Detection in Field-Caught Aedes aegypti Mosquitoes from Argentina Using NGS Technology. PLoS Neglected Trop. Dis..

[56] Chuchuy A., Rodriguero M., Alonso A., Stein M., Micieli M. (2024). Wolbachia Infection in Natural Mosquito Populations from Argentina. Parasitol. Res..

[57] Abbasi M., Yousefi S. (2025). Assessing Insecticide Susceptibility of Culex pipiens Linnaeus (Diptera: Culicidae) in the Aras River Basin: Implications for Disease Control. BMC Infect. Dis..

[58] Wang G., Hoffmann A., Champer J. (2024). Gene Drive and Symbiont Technologies for Control of Mosquito-Borne Diseases. Annu. Rev. Entomol..

[59] Zhang Y., Wang M., Huang M., Zhao J. (2024). Innovative Strategies and Challenges in Mosquito-Borne Disease Control Amidst Climate Change. Front. Microbiol..

